# A Rare Case of Paratesticular Sarcoma: A Case Report

**DOI:** 10.7759/cureus.58793

**Published:** 2024-04-22

**Authors:** Neil A Nunes, Abhi A Shah, Gopalakrishnan Murugan

**Affiliations:** 1 Department of Radio-Diagnosis, Sree Balaji Medical College and Hospital, Chennai, IND

**Keywords:** testicular swelling, testis cancer, orchidectomy, para testicular tumors, testicular mass

## Abstract

Paratesticular rhabdomyosarcoma (PRMS) is a rare and aggressive soft tissue tumour that can mimic testicular sarcoma on initial imaging studies, leading to diagnostic ambiguity and treatment delays. In this case report, we present the case of a 45-year-old male who came to our department and was evaluated under ultrasound imaging along with colour Doppler. The patient underwent further examination under a multi-slice CT machine, which provided additional information, and finally underwent a 1.5T MRI scan. After a provisional diagnosis was made, the patient underwent surgery, and the specimen was sent for histopathology and relevant immunohistopathological markers. This case underscores the diagnostic challenges posed by PRMS and emphasizes the need for a multidisciplinary approach involving radiologists, oncologists, and surgeons for timely diagnosis and optimal management. We discuss the clinical implications, imaging characteristics, differential diagnosis, and therapeutic considerations for PRMS to guide clinicians in similar diagnostic dilemmas.

## Introduction

Rhabdomyosarcoma (RMS) is an extremely aggressive mesenchymal tumour believed to stem from underdeveloped striated muscle. It is distinguished by the existence of cells demonstrating recognizable differentiation toward striated muscle, including rhabdomyoblast cells [1]. Approximately 20% of RMS cases originate in the genitourinary system. In adults, the pleomorphic variant is more common and is associated with a bleak prognosis [2].

The paratesticular region is a complicated anatomical region that encompasses structures like the spermatic cord, testicular trunks, epididymis, and residual parts such as appendices and remnants of the testis. Histogenetically, this area comprises a diverse range of epithelial, mesothelial, and mesenchymal elements. Consequently, tumours originating in this area create a varied collection of neoplasms exhibiting diverse behavioural patterns [3].

## Case presentation

A 45-year-old male patient presented to us in December 2023 with complaints of scrotal swelling for the last six months; the swelling gradually increased in size. The swelling was not tender to the touch. The swelling was approximately 20 cm in length, and the patient had a feeling of heaviness. The swelling was painless. On ultrasound screening, a lesion in the left hemi-scrotal region showed clear margins of the left testis from the tumor. The lesion showed good vascularity on the colour Doppler examination. The examination revealed no obvious regional lymph node enlargement.

**Figure 1:**
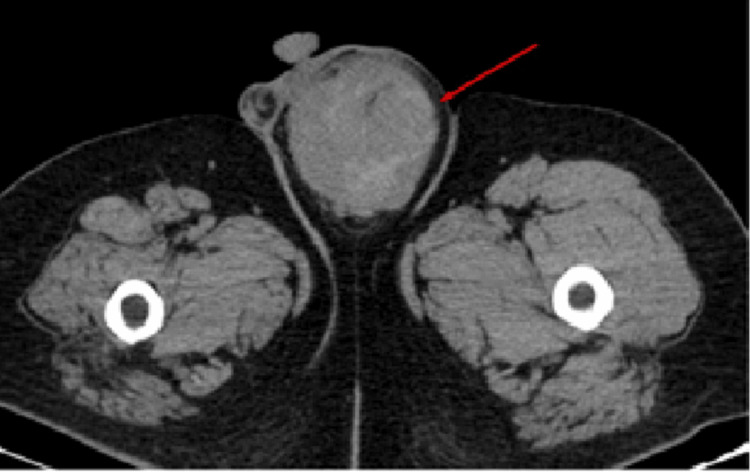
Axial section of non-contrast CT shows a well defined heterodense lesion in the left para testicular region (red arrow). No evidence of any calcific specs seen.

**Figure 2:**
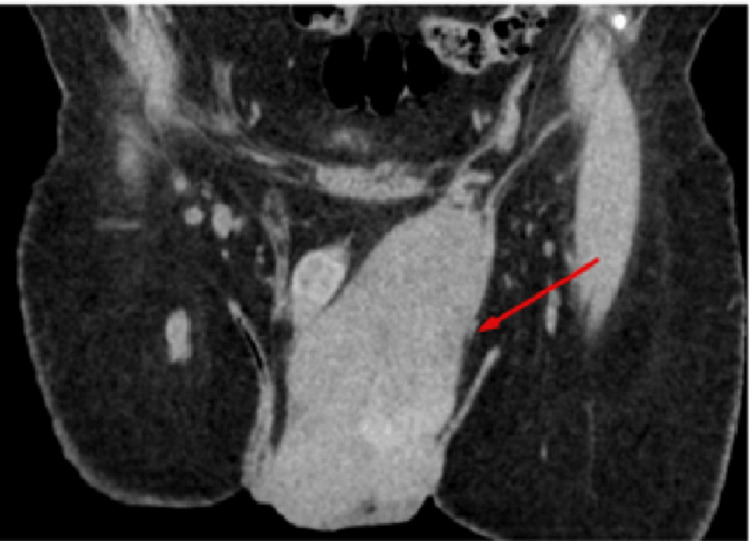
Coronal section of non-contrast CT shows its extension from left inguinal region to left hemiscrotum (red arrow).

**Figure 3:**
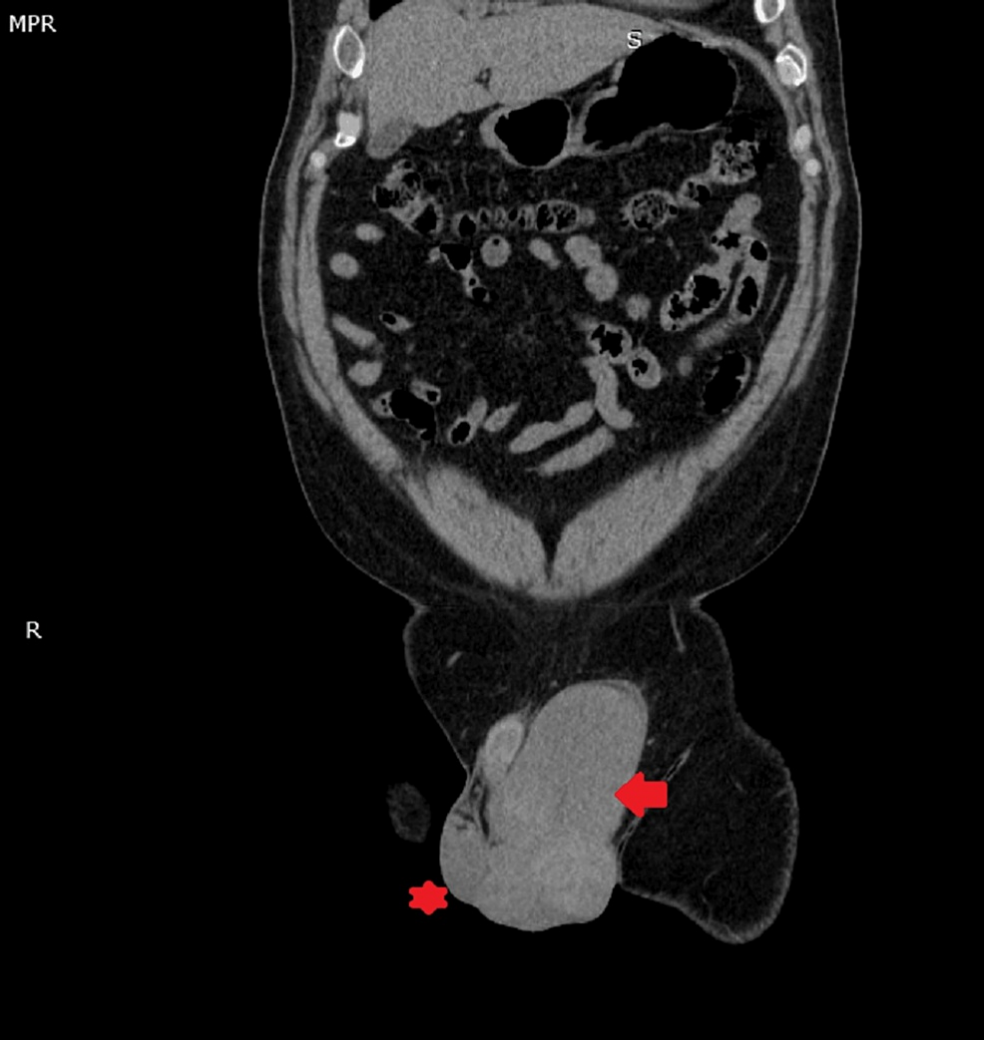
* (Asterix symbol) marks the location of the normal testis on the right of the patient, and the → (arrow head) symbol marks the location of the affected testis on the left of the patient. Scan showed is a coronal section of a non-contrast CT of the patient.

**Figure 4:**
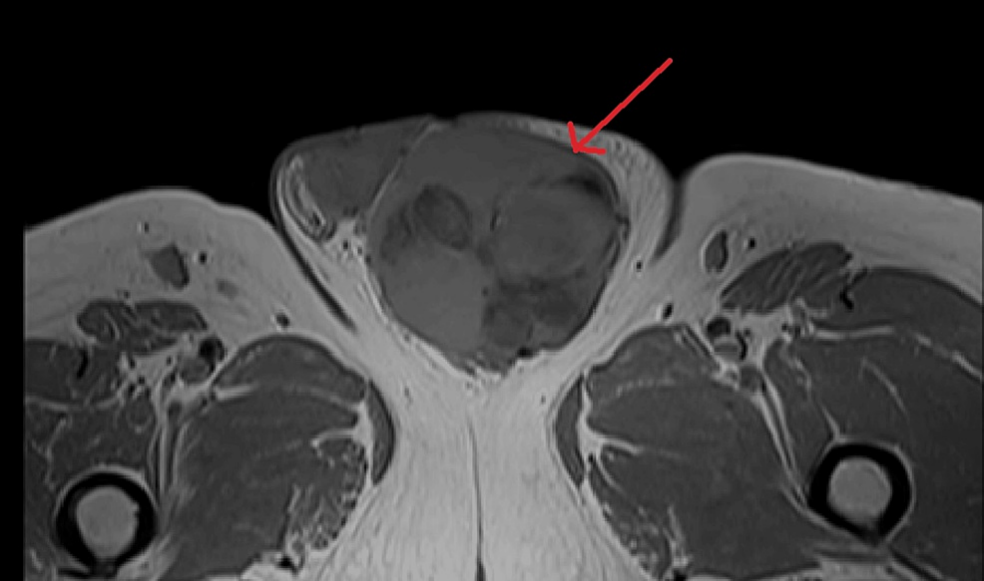
MRI T1 axial section show a multilobulated lesion with heterogenous signal in the left para testicular region.

**Figure 5:**
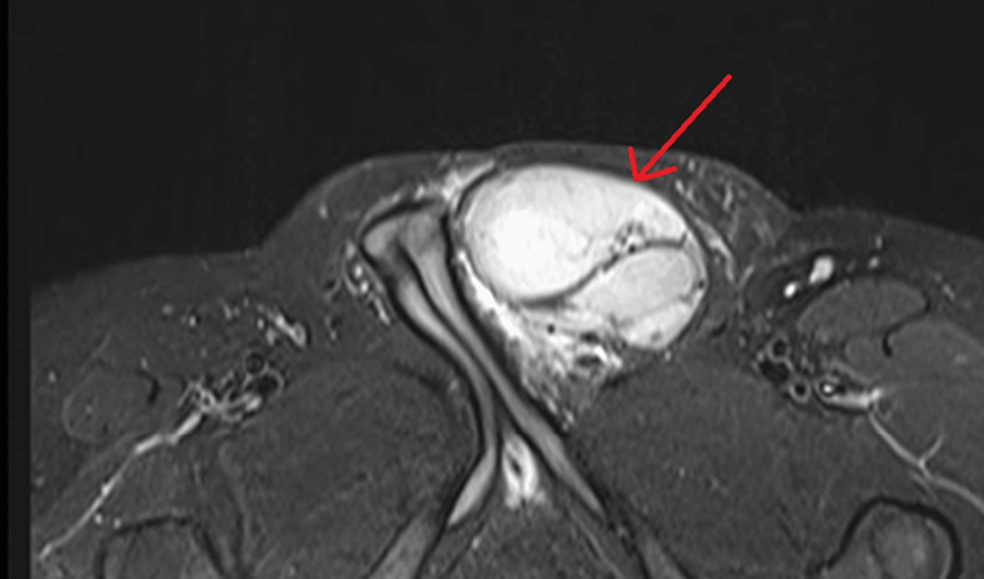
MRI T2 fat saturated axial section shows a multilobulated lesion with heterogenous signal in left para testicular region.

**Figure 6:**
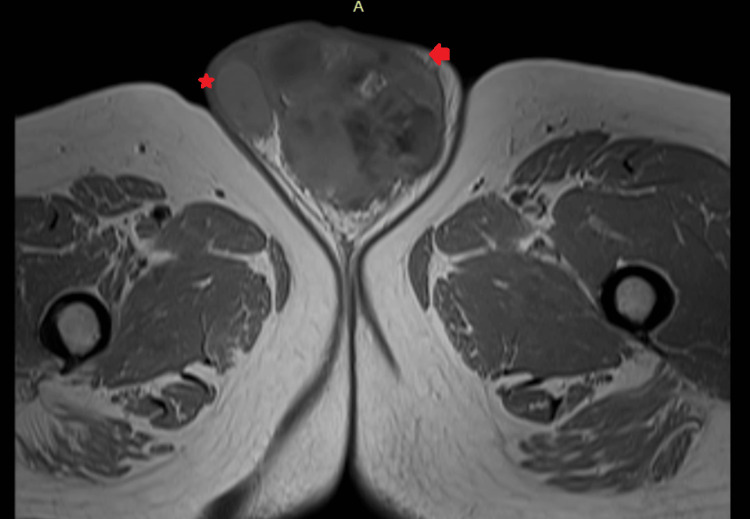
MRI T1 sequence showing "*" (asterisk symbol) as the unaffected testis on the right side of the patient, and "→" (arrow head symbol) as the affected testis on the left side of the patient.

**Figure 7:**
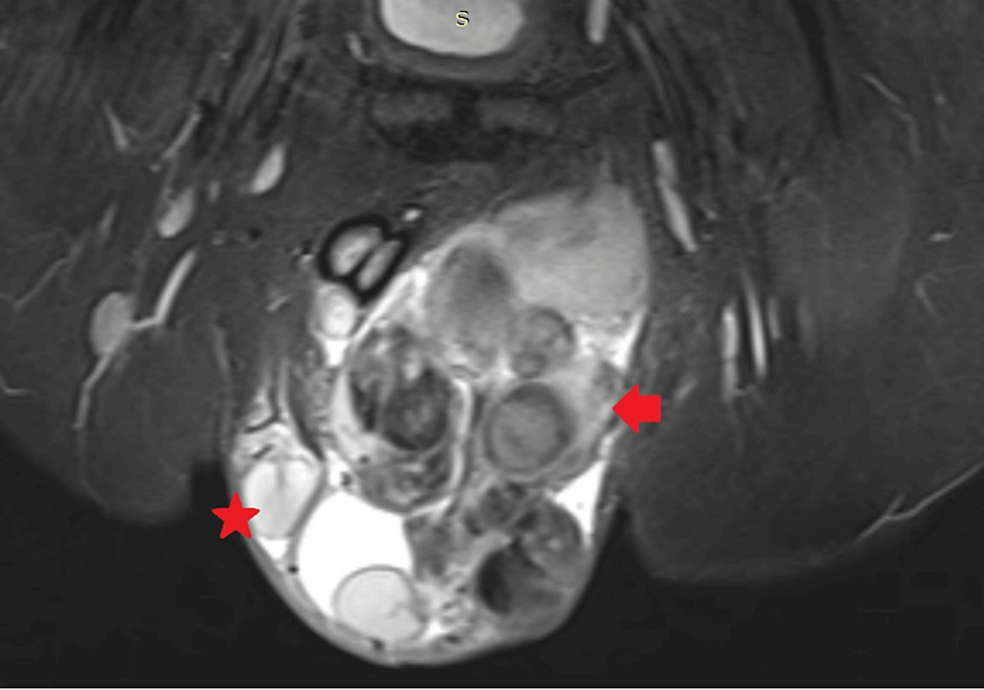
MRI T2 sequence in coronal section showing "*" (asterisk symbol) as the unaffected testis on the right side of the patient, and "→" (arrow head symbol) as the affected testis on the left side of the patient.

**Figure 8:**
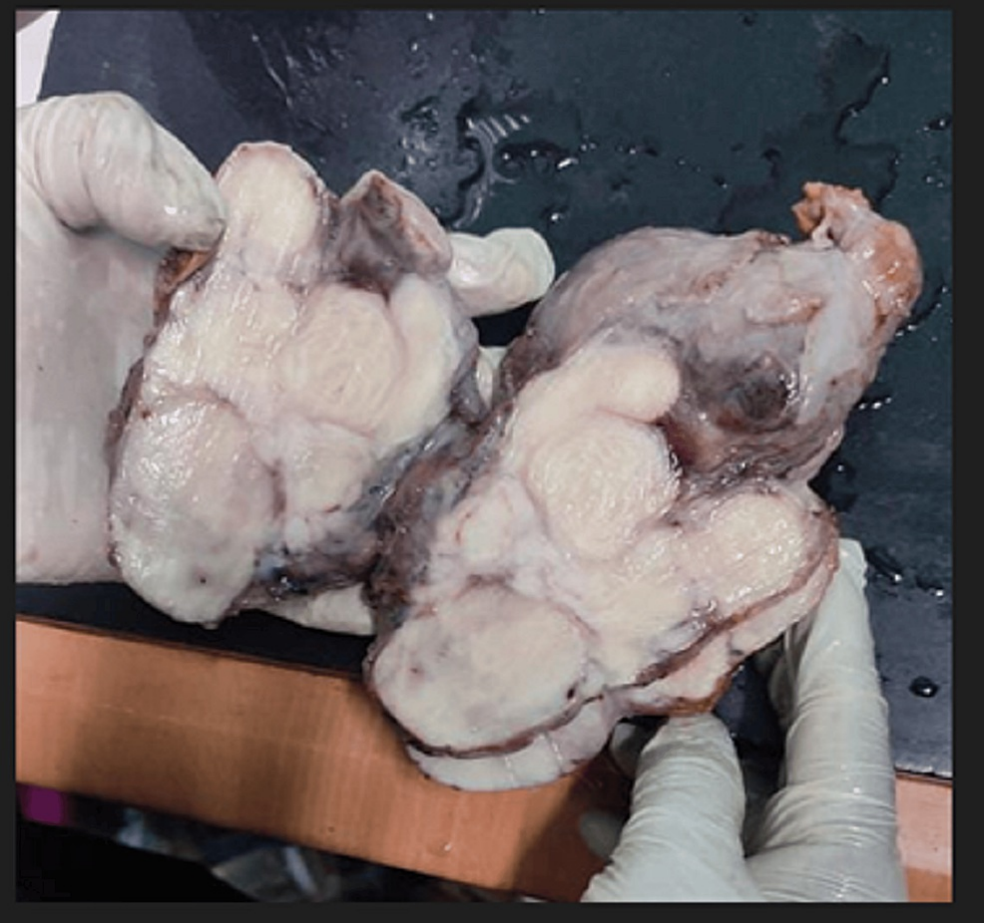
Gross surgical specimen of the multilobulated para-testicular lesion.

On computer tomography (CT) (Figures 1-3) and magnetic resonance imaging (MRI) (Figures 4-7), a heterogenous lobulated mass in the left scrotal sac extends from the left inguinal canal to the scrotal sac. No obvious calcification was seen. The patient was operated on with an excision of the lesion and a left-sided orchidectomy (Figure 8).

Based on the above radiological findings the differential diagnosis that could be made are: (a) adenomatoid tumor; (b) papillary cystadenoma; (c) fibrous pseudotumor or the scrotum. To differentiate between these and any other potential tumors, an orchidectomy along with histopathological examination was done and the findings are described as below.

Pathological report

Gross Findings

The specimen received was the left testis. The specimen measured ~15 cm × 10 cm × 10 cm with an attached cord that measured ~3 cm in length. The outer surface was encapsulated. The cut surface shows that the whole testis was replaced by a solid and firm tumor, which measured ~13 cm × 9 cm × 9 cm. The tumour appeared to infiltrate into the surrounding tissue in the form of nodules. The tumour involved the epididymis and was ~3 cm away from the resected cord margin. The cut surface of the cord appeared to be unremarkable.

Microscopic Findings

**Figure 9:**
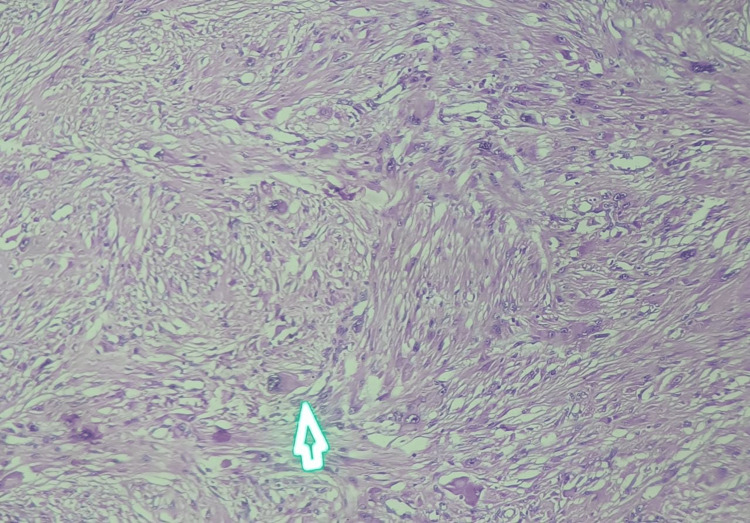
100× image of the observed tumour shows an ill-defined tumour comprising spindle-shaped cells arranged in sheets and fascicles. These spindle-shaped cells exhibit moderate nuclear pleomorphism.

**Figure 10:**
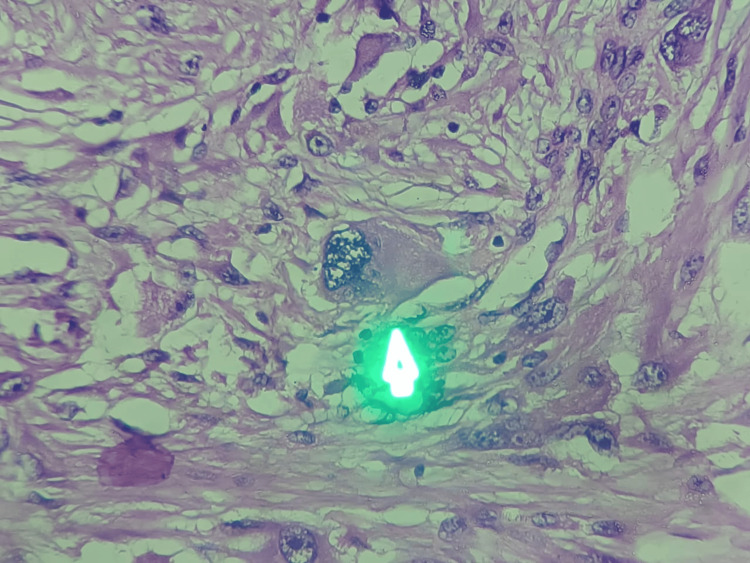
At 400× magnification, the tumour tissue section reveals large rhabdomyoblastic cells with eccentric, pleomorphic vesicular nuclei and deeply eosinophilic cytoplasm.

The section showed an ill-defined tumour comprising spindle-shaped cells arranged in sheets and fascicles (Figure 9). These cells show moderate nuclear pleomorphism, with areas of coagulative necrosis. A few epithelioid large cells are noted with a prominent vesicular nucleus, conspicuous nucleoli, and eosinophilic cytoplasm (Figure 10). The section also showed metaplastic bone formation and atypical mitosis. These tumour cells also infiltrated into the epididymis. Sections from the resected cord margin were histologically unremarkable.

The samples were collected and sent for histopathological examination and immunohistopathological study, which showed vimentin and CD-99 were strongly positive in tumour cells, desmin was focally positive, smooth muscle actin was negative in tumour cells, CD 34 and S100 were negative in tumour cells, and Ki 67 was 40%. The final diagnosis was pleomorphic rhabdomyosarcoma, histologic grade 3. No lymphovascular invasion was noted. No regional lymph node invasion was noted.

The patient had an uneventful hospital stay and was discharged on the second day after the operation (POD-2). As sections from the resected cord were clear and no lymphovascular invasion or regional lymph node invasion was noted, the patient was advised to be on strict follow-up. At present, the patient remains in follow-up with the urology and oncology departments.

## Discussion

Paratesticular sarcomas refer to a broad category encompassing tumours that develop within the scrotum but are not of testicular origin. This includes tumours arising in the epididymis, spermatic cord, tunica vaginalis, and other supporting structures. An estimated 70% of paratesticular tumours are benign and 30% are malignant [3]. Paratesticular rhabdomyosarcomas encompass various subtypes, such as embryonal, alveolar, and pleomorphic [4]. The majority of cases exhibit embryonal histology. A less common variant within the embryonal subtype is the spindle cell type, initially documented by Cavazzana et al. in 1992. Adult cases of spindle cell type constitute 3% of all rhabdomyosarcomas. It is worth mentioning that there are limited case reports describing paratesticular lesions in adults [4].

Typically, spindle cell-type paratesticular rhabdomyosarcoma is linked to a positive prognosis, exhibiting a 95% five-year survival rate [5]. Moreover, the occurrence of lymph node metastasis is less frequent in the spindle cell variant when compared to non-spindle cell variants (16% vs. 36%). [5]

Some radiological findings of RMS can include that, on CT, it may appear as a soft tissue density lesion, which may show some enhancement on contrast if administered. It may also show adjacent bone destruction [6]. On MRI, signal changes that may be seen on T1-weighted images include low to intermediate signal intensity, and the lesion may be iso-intense to adjacent muscle. On T2-weighted images, they may appear as hyper-intense lesions with prominent flow voids, particularly in the extremities of the lesions [6].

Given the rarity of para-testicular spindle cell rhabdomyosarcomas, our case was notably uncommon as it occurred in a 45-year-old man, a departure from the usual occurrence of spindle cell variants in childhood. Although instances of such tumours have been documented globally in countries like India, Japan, Turkey, and Syria, they remain considerably fewer in number compared to the more prevalent scrotal or testicular malignancies [7,8].

The use of scrotal ultrasound, CT scan, testicular tumour markers, and FNAC (fine-needle aspiration cytology) needle biopsy will narrow down the diagnosis. Surgical excision through an inguinoscrotal incision will be the treatment of choice [9].

Multidisciplinary approaches are typically advised due to the lack of a standardized treatment protocol. The primary surgical treatments include radical orchidectomy, hemiscrotectomy, and high inguinal cord dissection, along with inguinal lymph node dissection. Rhabdomyosarcoma (RMS) demonstrates sensitivity to chemotherapy, with the VAC protocol (vincristine, actinomycin-D, and cyclophosphamide) being commonly employed. Radiotherapy is recommended for addressing any residual microscopic tumor foci [10].

## Conclusions

Paratesticular rhabdomyosarcomas are uncommon tumours that exhibit concerning characteristics on ultrasonographic examination, such as enlarging solid masses with increased vascularity. The referring department should approach these findings with a heightened level of suspicion and include them in the list of potential differential diagnoses. Optimal management may involve a radical orchiectomy followed by adjuvant chemoradiation. Overall, embryonal spindle cell-type rhabdomyosarcomas are associated with a favourable prognosis.
